# Rho GTPase ROP1 Interactome Analysis Reveals Novel ROP1-Associated Pathways for Pollen Tube Polar Growth in Arabidopsis

**DOI:** 10.3390/ijms21197033

**Published:** 2020-09-24

**Authors:** Hui Li, Jinbo Hu, Jing Pang, Liangtao Zhao, Bing Yang, Xinlei Kang, Aimin Wang, Tongda Xu, Zhenbiao Yang

**Affiliations:** 1 CAS Center for Excellence in Molecular Plant Sciences/Shanghai Institute of Plant Physiology and Ecology, Chinese Academy of Sciences, Shanghai 201602, China; hujinbo@sibs.ac.cn (J.H.); zltdo@163.com (L.Z.); yangbing@sibs.ac.cn (B.Y.); kangxinlei@sibs.ac.cn (X.K.); tdxu@sibs.ac.cn (T.X.); 2 Center for Plant Cell Biology, Institute of Integrative Genome Biology, and Department of Botany and Plant Sciences, University of California, Riverside, CA 92508, USA; yang@ucr.edu; 3 Shanghai Institute of Plant Physiology and Ecology, University of Chinese Academy of Sciences, Beijing 100049, China; 4 School of Life Sciences, Jiangsu Normal University, Xuzhou 221116, China; jingpang@shsmu.edu.cn (J.P.); aiminwang@jsnu.edu.cn (A.W.); 5 FAFU-UCR Joint Center for Horticultural Biology and Metabolomics, Institute of Science and Technology, Fujian Agriculture and Forestry University, Fuzhou 350002, China

**Keywords:** ROP1 GTPase, protein interactome, polar tip growth, pollen tube, Arabidopsis

## Abstract

ROP (Rho-like GTPases from plants) GTPases are polarly localized key regulators of polar growth in pollen tubes and other cells in plants. However, how ROP GTPases are regulated and how they control polar growth remains to be fully understood. To gain new insights into ROP-dependent mechanisms underlying polar cell growth, we characterized the interactome of ROP1 GTPase that controls Arabidopsis pollen tube (PT) tip growth, an extreme form of polar cell growth. We established an efficient method for culturing Arabidopsis pollen tubes in liquid medium, which was used for immunoprecipitation/mass spectrometry-based identification of ROP1-associated proteins. A total of 654 candidates were isolated from the ROP1 interactome in Arabidopsis pollen tubes, and GO (Gene Ontology) classification and pathway analysis revealed multiple uncharacterized ROP1-dependent processes including translation, cell wall modification, post transcriptional modification, and ion homeostasis, in addition to known ROP1-dependent pathways. The ROP1-interactome data was further supported by the co-expression of the candidate interactors in highly mature pollen with PT germination and growth defects being discovered in 25% (8/32) of the candidate mutant genes. Taken together, our work uncovers valuable information for the identification and functional elucidation of ROP-associated proteins in the regulation of polar growth, and provides a reliable reference to identify critical regulators of polar cell growth in the future.

## 1. Introduction

The establishment and maintenance of cell polarity plays a pivotal role in growth, development, and survival of various organisms [1]. Tip growth is an extreme form of polar growth, showing cell expansion restricted to the apical domain, and this growth model is utilized for functional specification of cells with long tube-like shapes, such as pollen tubes for sperm delivery into the ovary, plant root hair elongation for nutrient foraging in soil, fungal hyphal growth for host invasion, and the extension of neuronal axons for signal transmission in animals [2,3,4].

Rapidly tip-growing pollen tubes are regarded as an excellent model for deciphering the regulatory mechanisms underlying polar tip growth in plants [5,6,7]. Pollen tubes are divided into the apical, subapical, and shank region, and various processes in the rapidly expanding apical region are precisely coordinated for the maintenance of rapid tip growth, such as cytoskeleton organization, endomembrane trafficking, exocytosis and endocytosis, calcium gradient, and signaling events [6,7].

Recent studies on plant female–male crosstalk indicated that the small GTPase ROP1 (ROP—Rho-like GTPases from plants) is an important hub in pollen tube (PT) to perceive cues from the female tissue through cell surface receptors, and initiates PT tip growth by transducing a signal to downstream effectors [8,9,10]. Meanwhile, the similarity of pollen tube growth patterns in vivo and in vitro indicated pollen tube tip growth is a self-organized regulatory system maintained by an autocrine signal [11], where the internal ROP1 signaling pathway plays a central role in orchestrating and maintaining the tip region and polar growth of PT through downstream effectors [5,7,12,13].

Polar growth mechanisms regulated by the Rho GTPases family involved in the establishment and maintenance of cell polarity was found to be highly conserved across eukaryotic kingdoms. Major advances from pollen tube studies also indicated that the Rho small GTPase, ROP1, acts as a molecular switch that can be directly turned on/off by the conserved GTPase regulatory proteins RhoGEFs (guanine nucleotide exchange factors) and RhoGAPs (GTPase-activating protein) in Arabidopsis [14]. The autoinhibition release of RhoGEFs is required for the conversion of the Rho GTPase from the GDP-bound inactive form into an GTP-bound active form via an interaction with kinase family members [8,15,16], with RhoGEFs polar distribution in pollen tube being maintained by the cytoplasmic kinases AGC1.5/AGC1.7 [17]. The RhoGAP REN1 localizes to the apical plasma membrane (PM) region for the downstream regulation of ROP1 activity via ROP1-dependent vesicular exocytosis [18].

Maintenance of the polar distribution of ROP1 in the apical PM is also essential for pollen tube tip growth. ROP1 polar distribution in the apical PM is dynamically modulated by GDI members (Rho guanine nucleotide dissociation inhibitor), which moves active ROP1 from the membrane to the cytosol [19,20,21,22]. The plant-specific RIP1/ICR1 plays a role in promoting ROP1 recruitment to the apical PM of pollen tubes [23]. Recently, in fast-growing and slow-growing pollen tubes, a novel WD40 protein, REN4, was reported as a gatekeeper that not only dynamically maintains the demarcation between the apical and lateral domains by active ROP1/REN4-mediated endocytosis in the subapical region, but also spatiotemporally fine tunes the ROP1 activity in apical PM [22].

The dynamic reorganization of the ubiquitously-distributed cytoskeleton was determined by the ROP interacting CRIB-motif (RIC) proteins, RIC3 and RIC4, in two antagonistic pathways: RIC3-induced elevation of Ca^2+^ leads to disassembly of RIC4-induced F-actin, leading to oscillating pollen tube tip growth [24]. Moreover, the cytoskeleton appears to be a key player in the feedback regulation to dynamically modulate ROP1 activity and its polar distribution in the pollen tube. For example, F-actin mediated vesicular exocytosis in the tip plays a fundamental role in establishing the domain of active ROP by involvement of the upstream regulators REN1/PRK2 secretion to apical PM [18,25]; actin-involved endocytosis was also reported to mediate ROP1 distribution in the apical PM region [22].

Evidently, ROP GTPase activity represents a key signaling hub coordinating polar growth. These molecular switches and their up- and down-stream components appear to be connected with additional signaling pathways underlying PT tip growth. However, the detailed molecular mechanism of ROP1 role in the regulatory systems of PT polar tip growth remains largely enigmatic.To elucidate the ROP1-dependent molecular mechanism for PT growth, we first established a method to promote PT germination in liquid medium from which PT can be easily collected. Following efficient pollen tube germination, we then carried out protein co-immunoprecipitation and proteomic assays in Arabidopsis PT, and initiated the screen and functional classification of ROP1-associated proteins in Arabidopsis PT. We found 654 candidate proteins using the mass spectrometry technique. Thirty percent of previously reported ROP1 interactors were also found in our results. More importantly, we found new candidates for ROP1-mediated pollen tube growth. The expression pattern assay further supported the function of these candidates and known interactors by showing that most interactors were preferentially expressed in pollen. Bioinformatic analysis revealed that ROP1 and these interactors were not only involved in the reproductive process, PT growth, cytoskeleton organization, but also in novel biological processes, such as translation, cell wall modification, post transcription modification, and ion homeostasis. Hence, our work provides a reliable reference for a protein interactive assay in PT. Moreover, this research will shed light on the self-sustained regulatory machinery underlying cell tip growth in other species.

## 2. Results

### 2.1. Isolation of ROP1-Associated Interacting Proteins from Arabidopsis Pollen Tubes

**Figure 1:**
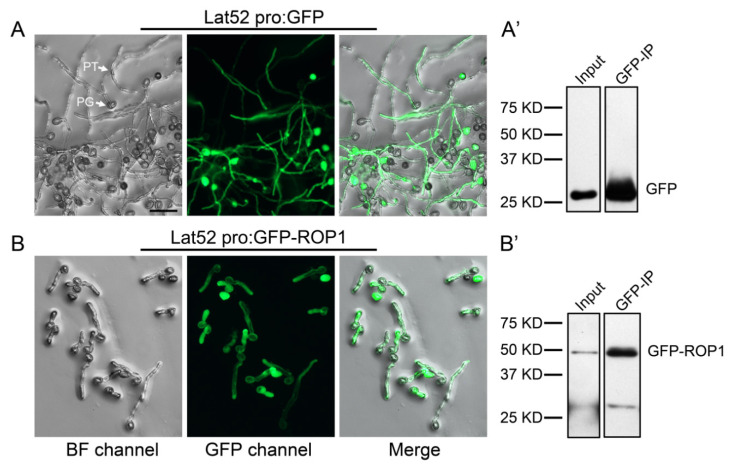
Pollen tubes of GFP (Green Fluorescence Protein) and GFP-ROP1 expressing transgenic lines. Mature pollen was collected from open flowers and cultivated on pollen germination medium for 4 h at 28 °C. Tubes of GFP labeled showed tip and polar growth (**A**), but the GFP-ROP1 expressing pollen tubes showed slightly depolarized growth (**B**). GFP (**A’**) or GFP-ROP1 (**B’**) proteins were precipitated from pollen tubes of the two transgenic line using GFP-Trap beads and were detected by Western blot using the anti-GFP antibody.

The expression and activity of the small GTPase ROP1 determines PT tip specification and polar growth in Arabidopsis [26,27,28]. In order to dissect the molecular mechanism of polar tip growth, we initiated the screening of ROP1 associated proteins by affinity purification using beads covalently conjugated with GFP (Green Fluorescence Protein) antibody in cultured Arabidopsis PT. ROP1 associated proteins were co-precipitated from Arabidopsis PT of a GFP-ROP1 overexpressing stable line, which exhibits slightly depolarized growth (Figure 1B) [28]. As a negative control, GFP beads were incubated with protein extracts of *Lat52 pro:GFP* expressing PT, which show standard tip growth on medium (Figure 1A). The specific binding between GFP beads and GFP or GFP-ROP1 fused protein were detected and verified by Western blot prior to identification using mass spectrometry (Figure 1A’,B’). Three biological replicates were included in both GFP-ROP1 and control groups. Relative to the GFP negative control, 654 ROP1 associated proteins were specifically isolated from GFP-ROP1-expressed pollen tubes (Table S1). Detailed information, including the accession number and coverage of all the identified proteins, is listed in Table S1.

### 2.2. Bioinformatic Analysis of ROP1-Associated Proteins in Arabidopsis

**Figure 2:**
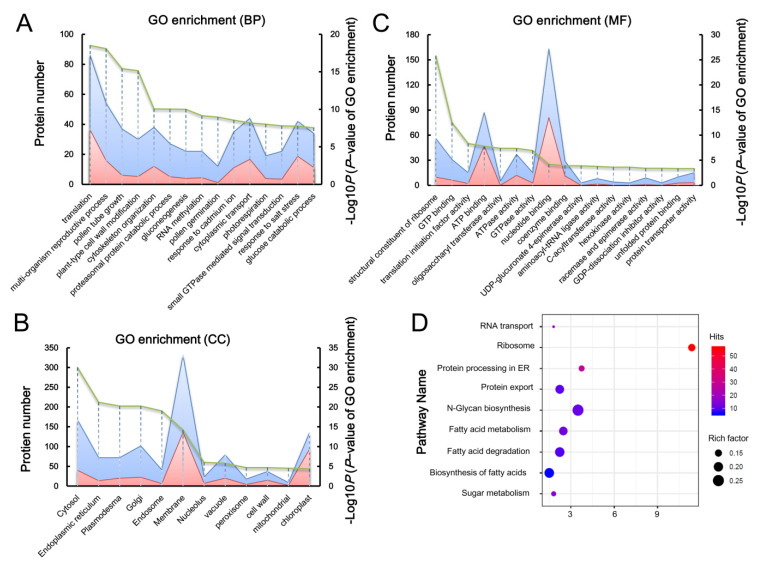
The GO and KEGG (Kyoto Encyclopedia of Genes and Genomes) classification of ROP1-associated protein in Arabidopsis pollen tubes. According to P value (green line), protein number (blue) and significance (pink), the 15 most significant biological processes (**A**), the 12 most significant molecular functions (**B**), and the most significant 12 subcellular localizations (**C**) and KEGG enrichment results (**D**) of ROP1-associated proteins were shown.

GO annotation provides general information on the basic function of proteins in three aspects of biological process (BP), cellular component (CC), and molecular function (MF). ROP1-associated proteins were thus subjected to functional classification by GO analysis. The analysis revealed that the most significant processes of ROP1-associated proteins included reproductive processes, pollen tube germination and growth, cell-wall modification, cytoskeleton organization, protein transport, and small GTPase-mediated signaling transduction (Figure 2A), which was consistent with previous reports of ROP1-mediated biological processes [16,21,24,25,29,30]. This further implied the reliability and accuracy of our immunoprecipitation and proteomics results for the ROP1 interactome. Additionally, we found that the top enriched GO term for ROP1-binding proteins is translation (Figure 2). We hypothesized that active translation is important and necessary for rapid tip growth of pollen tubes, with the regulatory role for translation being related to the cytosol-distributed ROP1, whose biological function in translation was unknown until now. Functional studies of these proteins will uncover novel regulatory mechanisms of ROP1-mediated PT polar growth.

GFP-fused ROP1 showed a ubiquitous distribution in the cytosol (GDP-bound inactive form of ROP1) and plasma membrane (GTP-bound active form of ROP1) of PT [22]. According to the cellular component (CC) terms from the GO annotation, the cellular localization of ROP1 interactors was mainly confined to the cytosol and ER (endoplastic recticulum), two sites for protein translation and processing (Figure 2B). This result was also consistent with the most highly enriched biological process (BP) terms of interactors involved in the translation process (*p* value < 0.0001). Subsequently, the classification of cellular components also revealed that most ROP1-associated proteins were mainly associated with membranes (331/621), where active ROP1 is specifically distributed in the PT (Figure 2B). This finding indicated that the membrane system plays a pivotal role in active ROP1-directed tip and polar growth of PT. Intriguingly, we also found that many ROP1 associated proteins localized to the Golgi, endosomes, and vacuole. We hypothesized that the specific localization of the ROP1-associated proteins possibly plays a role in the trafficking of ROP1 or its regulators (Figure 2B).

The top three enriched GO terms in the molecular function category for ROP1-associated proteins were structural constituents of ribosomes, GTP binding, and translation of initiation factor activity (Figure 2C). This is also in accord with the top term of translation in the biological process category for ROP1-interactive proteins in pollen tube. The other significant terms for the most abundant ROP1-binding proteins (163/621) were mainly found in the regulatory roles of nucleotide binding, including GTP and ATP (Figure 2C). Nucleotide binding of GTP or GDP plays a key role in the switching of the activation and inactivation status of ROP1. The significant enrichment of GTP binding activity in the molecular function classification suggested that the ROP1 interacting proteins may positively regulate ROP1 activity by catalyzing the transition of GDP to GTP. The maintenance of the dynamic balance of ROP1 activity is beneficial for the rapid pollen tube tip growth.

To gain further insight into the potential roles of ROP1-associated proteins, Kyoto Encyclopedia of Genes and Genomes (KEGG) analysis was performed to identify potential pathways involved. The KEGG assay showed that 9 metabolic pathways were significantly enriched (Figure 2D). Among them, the most significant terms were ribosome and protein processing in the ER, which are related to protein translation. Other significant terms included N-Glycan biosynthesis, fatty acid biosynthesis, metabolism and degradation, RNA and protein export, and amino sugar and nucleotide sugar metabolism. These results implied the existence of a coordinated network between ROP1 and its interactors in rapid tip growth through fast protein translation, processing, and metabolism.

### 2.3. Coexpression Assay of ROP1 and ROP1-Associated Proteins in Arabidopsis Pollen Tubes

**Figure 3:**
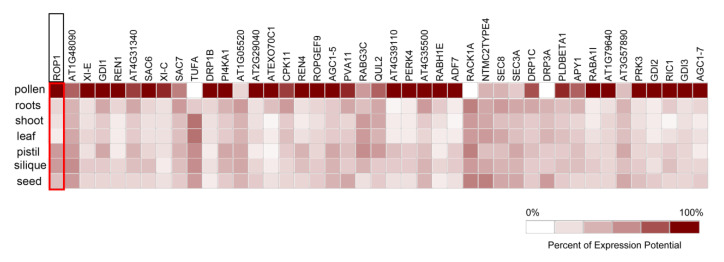
Analysis of expression levels of representative genes found to associate with ROP1 in different tissues of Arabidopsis. The proteins were ranked from left to right according to the detected unique peptide counts (Table S1) in the mass spectrum assay.

To compare the ROP1 transcriptional profile with its associated proteins, we performed the coexpression assay using Genevestigator. We found that ROP1 exhibits high specific transcription levels in mature pollen (Figure 3), which is consistent with the known ROP1 function in pollen tube germination and growth [16,21,24,25,29,30]. Among candidates, the expression profile of reported genes involved in the ROP1 signaling and represent genes for cytoskeleton, calcium signaling, vesicular trafficking, and phospholipid metabolism with detected peptides, ≥ 3 were analyzed. In the results, most ROP1-associated proteins from our study showed an expression pattern similar to ROP1 (Figure 3). However, according to the ranking of detected unique peptide counts, the highly enriched ROP1-associated proteins identified by MS detection do not always show high expression levels in mature pollen, such as TUFA, RACK1A, and DRP3A (Figure 3). It is possible that some proteins are pre-synthesized in pollen and may appear low at the transcript level [31]. Alternatively, the enrichment of candidates was depended on their affinity between ROP1 and its associated protein. Similarly, this case also fits with the expression profile assay of the top 400 ROP1 associated genes in our screening (Supplemental Figure S1).

### 2.4. Functional Assay and Confirmation of ROP1 Interactors in Arabidopsis Pollen Tube Tip Growth

To further verify our proteomics results of ROP1 interacting proteins, the ROP1 interactors confirmed by experimentation were selected from the STRING database. In total, 35 reported ROP1 interactors were identified in string database (Supplemental Figure S2), with recently reported ROP1 interactors (PRK3, AGC1.5, AGC1.7, and REN4), 30.77% (12/39) of these interactors were identified in our screen (Table 1). The canonical regulators of Rho GTPases—guanine nucleotide exchange factors (GEF), GTPase-activating proteins (GAP), and guanine nucleotide dissociation inhibitors (GDI)—are regulated similarly across eukaryotic species, and the upstream regulators of ROP1 GTPase including RhoGEF (RhoGEF9), RhoGAP (REN1), and RhoGDIs (SCN1, GDI2, and GDI3) members were all present in our results [18,19,20]. We also identified PRK3, a protein receptor kinase, which is involved in ROP1 signaling activation and interacted with GEF members in Arabidopsis pollen tubes [8]. Two functionally redundant cytoplasmic kinases, AGC1.5 and AGC1.7, were also identified and these are reportedly involved in ROPGEFs phosphorylation to maintain the polar localization of ROP-GTP in apical PM [17]. Three RICs (for Rop-interactive CRIB motif-containing proteins) were identified as ROP1-associated proteins in our screen [24,32]. The recently reported WD40 protein REN4, a rheostat to dynamically maintain the active ROP1 polar distribution in apical PM, and internalization into cytosol by endocytosis with lateral expanded active ROP1 in the subapical PM, was also found in our screen [22]. For 23 reported ROP1 interactors undetected in our work, we hypothesized that their low expression enrichment is relatively difficult to detect by IP-MS. To verify our hypothesis, the expression level of reported ROP1 interactors in pollen was further compared in the database of Genevestigator. We found most undetected reported ROP1 interactors showed lower expression level in pollen compared to that detected in our assay. Hence, interactors with high pollen expression and protein abundance were more easily isolated by IP-MS detection.

**Figure 4:**
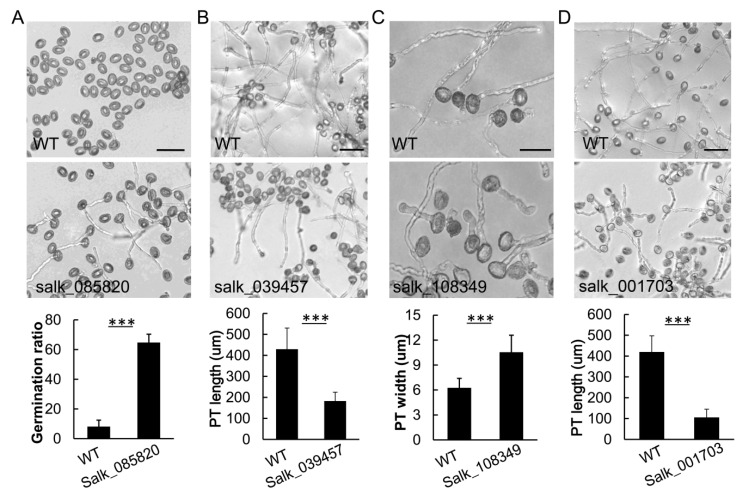
The pollen tube growth defects caused by the T-DNA insertions in four ROP1-associated proteins in Arabidopsis. The knockout line (Salk_085820) of the AAA-type ATPase family protein had promoted rates of pollen tube germination (**A**); The T-DNA insertion line (Salk_039457) in CHX28 led to the inhibition of pollen germination and shorter pollen tubes compared to wild type (**B**). The T-DNA insertion line (Salk_108349) for the EXOSTOSIN family protein caused swollen pollen tubes (**C**). The T-DNA insertion line (Salk_001703) for QUASIMODO2 LIKE 2 exhibited shorter pollen tubes (**D**). Scale bars = 30 µm in **A**, 50 µm in **B** and **D**, 20 µm in **C**. *** *p* < 0.001 (two-tailed T-test), n ≥ 50.

In addition to the above reported ROP1 interactors for pollen tube tip growth in *Arabidopsis*, we also isolated T-DNA insertion homozygous mutants of 32 additional ROP1 candidate interactors in our findings from the CS27943 mutant libraries (Table S2). The in vitro pollen tube germination and tip growth, typically defective in the mutants of ROP1 or its regulators, were analyzed in the 32 mutants. Among them, we found five lines showing defective phenotypes in pollen tube germination and growth. The five candidates encoded the KINKY POLLEN (KIP) protein (AT5G49680), exostosin family protein (AT2G20140), cation/hydrogen exchanger CHX28 (AT3G52080), AAA-type ATPase protein (AT2G02480), and QUASIMODO2 LIKE 2 (AT2G03480). The *kip* mutant had a short pollen tube phenotype consistent with a previous report [33] (data not shown). The promotion of pollen tube germination was caused by the knockout of the AAA-type ATPase protein (Figure 4A). Statistical analysis indicated that 70 ± 2.5% of the *atpase* pollen grains germinated after being cultured for 1 h *in vitro*, with only 10 ± 3.8% of the wild type pollen germinating (Figure 4A). The PT of *chx28* mutants not only showed inhibition of germination rate (35% ± 4.7%) compared to wild type (89% ± 2.3%), but also displayed shorter pollen tubes compared to wild type (Figure 4B). The T-DNA insertion mutant for the EXOSTOSIN protein had PT that exhibited swollen phenotype (Figure 4C). The *quasimodo2 like 2* mutant displayed shorter pollen tubes compared to wild type (Figure 4D).

Functional redundancy among members of the same gene family plays an essential role in protecting pollen tube growth from biotic and abiotic stress to ensure successful plant reproduction. Further investigation demonstrated that several candidates among the 38 detected ROP1 interactors also showed functional redundancy with their homologues for PT germination and tip growth in *Arabidopsis.* Only the double mutant of *AtAPY1* and *AtAPY2* showed a complete inhibition of pollen germination [34]; the PT of *mlo5mlo9* were twisted and piled-up after sensing ovular cues [35]. Furthermore, mutants without optimal T-DNA insertion sites are unsuitable for phenotype observation. The *syp2* mutant with an insertion in coding region showed pollen tube germination defective [36]. However, there was no obvious defects in the line Salk_014614C with a T-DNA insertion in an intron in the CS27943 library in which only homozygous mutants were included. Meanwhile, artifacts possibly exists among 654 screened associated proteins for ROP1 signaling pathway in pollen tube due to filter conditions or others specific problems. Hence, the interactor and regulatory mechanism between ROP1 and these candidates still need to be further investigated in the future.

### 2.5. Protein Network Analysis of ROP1–Associated Proteins

**Figure 5:**
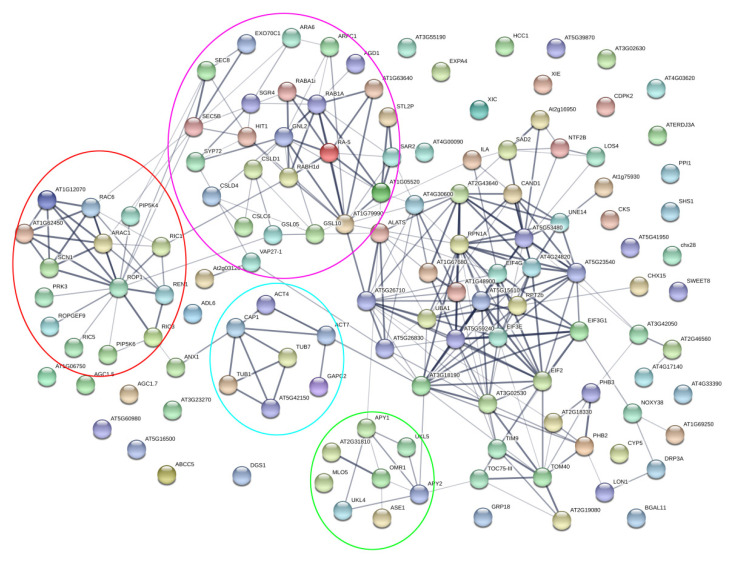
A putative network was assembled among 130 ROP1-associated proteins. The 130 proteins included members of multi-organism reproductive processes, pollen tube growth, plant-type cell wall modification, cytoskeleton organization, and intracellular transport based on the classification of biological processes.

Based on our screened candidates derived from the protein complexes with ROP1 in Arabidopsis pollen tubes, we hypothesized that an interactive network amongst these proteins can be assembled. To fully explore interactions among ROP1-associated proteins, a putative protein–protein interactive network was generated among 130 members, including the five BP classifications of multi-organism reproductive process, pollen tube growth, plant-type cell wall modification, cytoskeleton organization, and cytoplasmic transport. We found that 78% (102/130) of elements were part of complex interactions, and were tightly concentrated in five clusters (Figure 5). Some recently reported proteins such as AGC1.5 and AGC1.7 have not yet been linked with its interactor, GEF members, in the STRING database (Figure 5). Hence, it is possible that more ROP1-associated proteins in our screen showed interactions to some extent.

From Figure 5, it is apparent that the ROP1 cluster can be formed between ROP1 and its interactors such as RIC family members (RIC1, RIC3, and RIC5), RhoGAP REN1, RhoGDI (SCN1, GDI2 (AT1g12070), and GDI3 (AT1G62450)), RhoGEF9, PIPK kinases (PIP5K4 and PIP5K6), and receptor kinase PRK3 (red circle in Figure 5). Additionally, the ROP1 cluster showed links with vesicular trafficking thorough interactions between ROP1/RAC1/RAC6 and SEC family members (pink circle in Figure 5), which is consistent with the role of ROP1 in the exocytosis of secretory vesicles [25,37]. ROP1 is also linked with cell wall modification by its interaction with cellulose synthesis-like protein (CSL) and glucan synthesis-like protein (GSL5) family members (pink circle in Figure 5). We also identified RIC1 from this network analysis, which is involved in cytoskeleton organization and is linked with RAB family members, which play important roles in vesicular trafficking.

Currently it is unclear how RICs regulated a cytoskeleton structure. From our assay, we found that RIC3′s role with the cytoskeleton is possibly linked with the ANX1-CAP1-Actin4/7 pathway (blue circle). Furthermore, Actin7 interacts with TCP1 family member, At3g18190, which encodes chaperonin and plays a role in the folding of actin and tubulin. The regulators APY1/2 were interwoven with endomembrane trafficking and protein degradation via linkage with Sec23 and ubiquitin activating enzyme E1 (UBA1) in pollen tubes. The data indicated that ROP1 signaling is interwoven with other biological processes in pollen tubes, including downstream events such as vesicular trafficking and cytoskeleton organization. Hence, it is still to be verified whether other elements in our screen interacted with the ROP1 signaling pathway.

## 3. Discussion

The roles of the small GTPase ROP1 in governing pollen tube tip and polar growth were first established over 20 years ago [26,27]. In addition to the classical GTP-GDP cycling regulated by GEF/GAP, it is becoming increasingly clear that ROP1 signaling is also regulated by a wide range of mechanisms. Recent findings illustrate that ROP1 functions as an internal signaling hub in PT and mediates female–male communications during plant sexual reproductive development [8]. Proteins forming a complex with ROP1 is emerging as a key factor in the regulation of spatiotemporal ROP1 activation and polar distribution in PT. To further understand the ROP1-involved regulatory mechanisms for cell polar growth, we performed a proteomics analysis of the ROP1-related interactome in Arabidopsis pollen tubes by immunopreciptation. GFP antibody conjugated beads specifically bound to GFP and GFP-infused ROP1 from protein extracted from pollen tubes (Figure 1A’,B’), with 654 ROP1-specific binding candidates being identified from Arabidopsis PT. Besides the reported regulatory mechanisms such as GDP/GTP cycling, receptor or cytoplasmic kinases, cytoskeleton organization, and exocytosis pathways, our research revealed previously uncharacterized pathways that interact with ROP1 for PT self-sustained growth.

### 3.1. Cell Wall Modification and ROP1 Signaling

Plant cells are enveloped by a complex network of polymers-constituting the cell wall, which determine cell-shape and growth. To assure cell expansion without compromising cell integrity during pollen tube growth, a precise dynamic balance between cell wall structure and turgor pressure-driven cell expansion is crucial in the tip region. In response to various external signals, the changing cell wall structure is sensed by the RLK-ROP signaling pathway, which is instrumental for the adjustment of polar growth in pollen tubes [38]. However, little is known about the feedback regulation mechanism of how intercellular ROP1 signaling instructs cell wall formation and modification. Pectin is the major component of the cell wall in the tip of PT across plant species, and the highly methylesterified soft pectin and demethylesterified stiff pectin display regional distributions in the growing apex and the shank of PT, respectively [39,40,41,42]. The methylesterified and demethylesterified modification of apical pectin is mainly regulated by apical located pectin methylesterified enzyme (PME) and PME inhibitor (PMEI) [40]. Intriguingly, several highly expressed PMEIs (AT3G06830, AT3G62180, AT5G27870, and AT1G54620) in mature PT were identified as ROP1 interactors, but no PMEs were identified among the ROP1-associated proteins in our assay. Whether apical PMEI is inhibited by active ROP1 in fast growing PT tips or whether PMEI activity determines the dynamic conversion between stiff and soft pectin in the apex for slow or fast tip growing remains to be determined. The interaction of regulatory networks between ROP1 and PMEIs will be helpful in illustrating the ROP1 signaling mechanism underlying cell wall modification for PT polar growth.

### 3.2. Post-Translational Modifications and ROP1 Signaling

GTP-bound active form of Rho GTPase plays a pivotal role in various cellular processes via its interaction with a range of downstream effectors. The regulatory mechanism of Rho GTPase mediated by canonical GEF, GAP, and GDI is conserved across species, and these upstream regulators are often reported to result in GDP–GTP exchange of Rho GTPase, for example, the phosphorylation of RhoGEF members by PRK2 or AGC1.5 was demonstrated to lead to ROP1 activation in Arabidopsis pollen tube [16,17]. Hence, the dynamic balance of phosphorylation among GEF, GAP, and GDI plays a pivotal role for ROP1 signaling.

It has become apparent that the activity and distribution of Rho GTPases are controlled by post-translational modifications such as lipid modification, phosphorylation, ubiquitylation, and sumoylation [43]. The subcellular localization of Rho GTPases largely relies on lipid modifications such as palmitoylation and myristoylation. Furthermore, phosphorylation has diverse effects on RHO GTPase signaling, and was found to be involved in the GTP/GDP recycling, protein subcellular localization, sequestration from PM, and protein degradation [43]. In *cytotoxic lymphocytes*, RhoA GTPase was demonstrated to be phosphorylated by cyclic nucleotide-dependent protein kinase A (PKA) and PKG, with the phosphorylated RhoA having enhanced binding with GDI, which results in its withdraw from the plasma membrane [44]. The phosphorylation of ROP1 for pollen tube growth remains unclear. Although some kinases were identified in our screen, it remains to be investigated whether ROP1 phosphorylation was involved in its interaction with GEF, GAP, GDI, or its effectors.

Ubiquitylation is important for modulating protein levels by the 26S protease degradation system. Ubiquitin-activating (E1), ubiquitin-conjugating (E2), and ubiquitin ligase (E3) enzymes were required for the covalent attachment of ubiquitin polypeptides to target proteins. Within the E3 ligase superfamily, cullin RING ligases (CRLs) are significant in plants because they are coupled with numerous aspects of eukaryotic growth and development. A major mechanism modulating CRL activity is rubylation and derubylation, which is mediated by CULLIN-ASSOCIATED NEDD8-DISSOCIATED 1 (CAND1) in Arabidopsis [45,46]. We found that the PM distributed CAND1 (AT2G02560) had a highly specific affinity with ROP1 in PT (Table S1). Moreover, the ubiquitin-activating enzyme 1(AT2G30110), ubiquitin-conjugating enzyme (AT1G51730), ubiquitin-protein ligase 2 (AT1G70320), and ubiquitin 6 (AT2G47110) were found in the same protein complex as GFP-ROP1 in PT. This implied that ubiquitylation plays a regulatory role in protein turnover and PM distribution of active ROP1 in PT.

### 3.3. Material Transport and ROP1 Signaling in Pollen Tube

ABC ATP binding cassette (ABC) transporters constitute one of the largest protein families in plants and play a prominent role in a wide range of biological processes through the involvement in transporting complex organic materials, such as hormones, cell wall components, ions, or secondary metabolites [47]. The phylogenetic analysis of ABC proteins indicates that there are 8 subfamilies in Arabidopsis [47]. In our screen, 4 ABC proteins (AT5G60790, AT4G01660, AT5G60740, and AT5G24810) were found to be associated with ROP1 in PT. AT5G60790 was classified as a member of ABCF and likely functions in processes other than transport, as is the case for the yeast and human orthologs, which participate in ribosome recycling and translational control. ABCG28 (AT5G60740) was recently reported to be involved in ROS homeostasis in the PT tip region, and ABCG28 was localized to secreted vesicles and promoted PT tip elongation [48]. It remains to be verified whether ROP1 regulates ABCG28-included vesicular exocytosis and is involved in the ROS homeostasis in the tip region of PT. ABCA1 appears to be orthologous to the mammalian ABC1, and two ABCA1 members, AT4G01660 and AT5G24810, which co-precipitated with ROP1 in PT. The substrates transported by ABCA members were not clear until now, and whether their function is required for ROP1 signaling in PT will be investigated in the future.

Ion homeostasis plays an essential role in PT tip growth: ions, such as calcium, potassium, and H+, show dramatically polarized cytosolic gradients in the tip region, and their channels or transporters are required for PT tip growth [49,50,51]. Calcium displays a gradient in the PT tip region and is regulated by ROP1 downstream effector RIC3, therefore ROP1 activity oscillation is ahead of Ca^2+^ oscillation in directing PT growth. In our assay, transporters required for the flux of cation (AT2G22950), potassium (AT4G23640), phosphate (AT3G54700), magnesium (AT1G61790), and sulfate (AT1G22150) were also found. It will be interesting to investigate the regulatory mechanism between ROP1 signaling and transporter/channel activity, and whether these ions exhibit a polar distribution in the tip region of PT, which is still to be determined. Except for molecular material trafficking by the ABC family and ion transporters, other type of transporters were found for transmembrane trafficking of nutrients and energy, such as STP4&STP9 (AT3G19930 and AT1G50310) for sucrose and AT1G71680 for amino acids.

### 3.4. ROP1 Signaling and Endocytosis

Exocytosis of secretory vesicles is critical for pollen tube expansion by supplying membrane, cell wall components, and other material to regions exhibiting polar growth. The feedback regulation mechanism between ROP1 activity and vesicle exocytosis in apical region of pollen tube has been widely investigated [42,52]. ROP1 interacts with vesicular components of the Sec family for vesicular trafficking, docking, and fusion with the apical PM. Conversely, ROP1 inhibitors or activators are transported to the apex PM to precisely modulate ROP1 activity [18,25]. Subsequently, to maintain the dynamic balance of PT tip growth, extra membrane from vesicular secretion is recycled during endocytosis in the uptake of extracellular matrix components and PM polarization maintenance. Currently, the understanding of the mechanism of endocytosis regulating pollen tube tip growth is very limited. Recently, we demonstrated that laterally expanding ROP1 in the apex region was internalized in the subapical region via an interaction with REN4, with REN4 then conjugating with the ENTH adaptor protein EAP1 for clathrin-mediated endocytosis and dynamic maintenance of ROP1 polar distribution in the apex PM region [22]. In our assay, REN4 was also identified as a ROP1 interactor (Table 1). Other members of the ENTH family were also reported to be involved in endocytosis and the polar distribution ANX receptor kinase, which are upstream regulators of ROP1 signaling for guided pollen tube growth in vivo [53]. Meanwhile, the endocytic components dynamin were identified in our assay (AT3G61760, AT1G14830, AT4G33650, AT1G59610, and AT1G10290), but the detailed functions of these proteins has not been elucidated in PT. Further research is required to fully understand the relationship between ROP1 signaling and endocytosis.

## 4. Material and Method

### 4.1. Plant Materials and Pollen Tube Cultivation

All Arabidopsis materials were grown in greenhouses under long-day conditions (16 h light/8 h dark) at 23 °C, and 4–5-week-old plants were used, as younger or older plants produce less viable pollen. Open flowers were collected in 1.5-mL microfuge tubes using forceps, The microfuge tubes containing flowers filled with liquid pollen tube germination medium (18% sucrose, 0.01% boric acid, 1 mM CaCl_2_, 1 mM Ca(NO_3_)_2_, 1 mM MgSO_4_, pH 6.4) were capped, and vortexed vigorously for 5–6 min to release the mature pollen grains. The liquid mixture was then transferred into a new microfuge tube, and the mature pollen grains precipitated by centrifugation at 8000 rpm for 1 min.

Pollen tube germination and growth: The mature pollen collected from open flowers was resuspended using pollen germination medium (pollen density is about 5 × 10^3^ grain/μL), and a 20 µL drop dispensed onto a glass slide. The slide was then inverted and incubated for 3 h at 28 degree and 100% humidity.

Collecting pollen tubes: After checking the stage of pollen tube germination and growth in the hanging-drop cultivated medium under a microscope, the medium with germinated pollen tubes were collected using 200-µL pipettes pooled into a 1.5-mL microfuge tube. The microfuge tube containing the PT was centrifuged at 1000 rpm for 1 min, and the supernatant was carefully removed. The PT accumulated at the bottom of the tube and were ready for protein extraction. Collected pollen tubes can be stored at −80 °C.

### 4.2. Protein Extraction and Affinity Purification of Target Proteins

Protein extraction of pollen tubes: collected pollen tubes were gently dissolved in 200 µL NEB buffer (20 mM HEPES, pH7.5, 40 mM KCl, 1 mM EDTA) that contains 1 mM PMSF and the protease inhibitor cocktail. Once the pollen tubes were completely suspended, 200 µL NEB buffer containing 1% Triton X-100 was added and mixed well. The solution was then incubated on a rotating wheel for 15–30 min at 4 degree for protein extraction. The protein extracts were spun at 20,000 *g* for 15 min at 4 °C in a micro-centrifuge.

Immunoprecipitation of target protein: the protein extraction from PT was used for target protein binding assays using NEB buffer pre-washed GFP-Trap Agarose beads (gta-100, Chromotek, Planegg, Germany). The protein extraction and bead mixture was rotated for 2 h at 4 °C to allow the precipitation of the target protein and any associated protein complexes. To remove the unspecific protein bound to antibody-conjugated beads, the centrifuged protein-bead complex was washed three times using NEB buffer with 0.1% Triton X-100.

### 4.3. Western Blots Assay

To detect the specific and efficiency of immunoprecipitation, 1/10 (10 µL) beads as Western blot (WB) samples were taken out from total IP beads with the bound target proteins. WB samples were firstly reduced and denatured by boiling in SDS buffer at 100 °C for 5 min, then loaded and isolated in SDS-PAGE gel. Next, the isolated proteins in gels were transfer into PVDF membrane using Trans-Blot^®^SD semi-dry Electrophoretic Transfer Cell (Bio-Rad, Hercules, CA, USA) at 30 V for 25 min. For antibody staining, after 1 h blocking in blocking buffer (TBST buffer with 5% nonfat milk) at room temperature, the PVDF membrane with proteins was incubated overnight at 4 °C in the blocking buffer with 1:5000 diluted anti-GFP polyclonal antibody (PABG1-100, Chromotek). Then, washing the membrane using TBST for 3 × 5 min, and incubate the membrane in blocking buffer with the 1:5000 dilution of Goat Anti-Rabbit HRP conjugated secondary antibody (ab205718, Abcam, Cambridge, UK) at room temperature for 1 h. Wash the membrane three times again using TBST, 10 min each. For signal development, the membrane was covered with peroxide solution and the luminol/enhancer solution (SuperSignal West Femto, Thermo Fisher), then an image was acquired using a darkroom development instrument (DELIGHT-2010).

### 4.4. Identification of ROP1-Binding Protein by LC MS/MS

The ROP1-bound proteins were subjected to trypsin digestion (in-solution tryptic digestion and guanidination kit, Thermofisher Scientific, Waltham, MA, USA) and purified using a C18 column tip.

One ug peptide mixture was loaded onto a reverse-phase trap column (Thermo Scientific Acclaim PepMap100, 100 μm × 2 cm, nanoViper C18, Waltham, MA, USA) connected to a C18 reversed-phase analytical column (Thermo Scientific Easy Column, 10 cm long, 75 µm inner diameter, 3 μm resin) in buffer A (0.1% formic acid) and separated with a linear gradient of buffer B (84% acetonitrile and 0.1% formic acid), at a flow rate of 300 nL/min controlled by IntelliFlow technology. The total analysis time for each sample was 70 min. Mass spectrometry analysis was performed using the Orbitrap Fusion mass spectrometer (Thermo Scientific) that was coupled to Easy nLC (Proxeon Biosystems, now Thermo Fisher Scientific) for 70 min. Data dependent acquisition with full scans in the 350–1500 m/z range was carried out using an orbitrap mass analyzer at a mass resolution of 120,000. Most intense precursor ions were selected in the top speed data dependent mode with a maximum cycle time of 3 s. Peptides with charge 2–6 were selected and dynamic exclusion time was 30 s. Precursor ions were fragmented using higher-energy collision dissociation (HCD) and MS/MS ions were detected using the orbitrap at a mass resolution of 30,000.

### 4.5. Data Processing and Analysis

The MS data were analyzed using the Maxquant software (version 1.6.4.0) against the Arabidopsis database Araport11 (48,359 total sequences, downloaded from the TAIR website: www.arabidopsis.org). The following parameters were set. Cys alkylation: Iodoacetamide; Dynamic Modification: Oxidation (M), Acetyl (Protein N-Terminus); Static Modification: Carbamidomethyl (C); Enzyme Name: Trypsin (Full); Max. Missed Cleavage Sites: 2; Precursor Mass Tolerance: 10 ppm. Both the proteins and PSMs were filtered by FDR < 0.01. Proteins presented in all 3 biological replicates were considered as the ROP1 associated proteins in our research after excluding the negative control. More specifically, we chose the average unique peptide > 1 in the 3 biological replicate as further criteria.

### 4.6. Bioinformatic Analysis

Functional classification of the ROP1-bound proteins was performed by the R package of topGO based on the Gene Ontology database. The Kyoto Encyclopedia of Genes and Genomes (KEGG) database (http://www.kegg.jp/kegg/) was used to predict the pathways involving the ROP1-bound proteins. The p-value was used to evaluate the significance level of protein enrichment under each GO term and the KEGG pathway against the background. Protein enrichment with a *p*-value < 0.05 was considered to be significantly enriched against the background. Protein–protein interaction network analysis was performed in the STRING database (http://string-db.org/, version 11.0) based on the active experiment resources and medium confidence score.

## 5. Conclusions

In summary, the current understanding of the regulatory mechanism of Rop GTPase signaling in cell tip growth is very limited. For the establishment and maintenance of ROP1 activity and polarized distribution in rapid tip growing PT, the highly dynamic modification or interaction between ROP1 and its associated proteins are critical. Our results reveal novel and extensive ROP1 signaling pathways for pollen tube tip growth through a combination of an efficient method for Arabidopsis pollen tube culturing in liquid medium and the IP-MS technique. Moreover, except for the known ROP1-dependent pathway, most ROP1-associated candidates had high expression levels in mature pollen and were involved in PT tip growth. Taken together, our work will provide an important resource to uncover key regulators for cell tip growth.

## Figures and Tables

**Table 1:** The identified reported ROP1 interactors and pollen tube regulators identified in the screen.

Gene_ID	Protein Name	Function	Reference
AT3G07880	SCN1	Remove of membrane-distriubtion ROP1	Feng et al., 2016
AT4G24580	REN1	Deactivation of ROP1	Hwang et al., 2008
AT1G04450	RIC3	mediated ROP1-calcium signaling	Gu et al., 2005
AT2G26490	REN4	Endocytosis of PM-localized active ROP1	Li et al., 2018
AT4G13240	ROPGEF9	Activation of ROP1	Gu et al., 2006
AT3G23380	RIC5	ROP1-depended actin dynamic	Wu et al., 2001
AT1G12070	GDI2	Remove of membrane-distriubtion ROP1	Hwang et al., 2010
AT2G33460	RIC1	ROP1-mediated actin dynamic	Zhou et al., 2016
AT1G62450	GDI3	Remove of membrane-distriubtion ROP1	Feng et al., 2016
AT3G42880	PRK3	activate GEF1&12 for ROP1 singaling	Takeuchi et al., 2016
AT3G12690	AGC1.5	activate GEF1&12 for ROP1 singaling	Li et al., 2018
AT1G79250	AGC1.7	Reduance with AGC1.5 for ROP1 singaling	Zhang et al., 2009
AT5G49680	KINKY POLLEN	Short PT	Procissi et al., 2003
AT3G04080	AtAPY1	Inhibition of PG	Iris, et al., 2003
AT1G20080	SYT2	Inhibition of PG and short PT	Wang, et al., 2015
AT2G33670	MLO5	PT twist	Guo, et al., 2020
AT2G01540	Ca^2+^-dependent lipid-binding Protein	PT rupture	Wang, et al., 2018

(PM: plasma membrane, PT: Pollen tube, PG: Pollen germination).

## Supplementary Materials

Supplementary materials can be found at https://www.mdpi.com/1422-0067/21/19/7033/s1. Table S1. The Proteins that showed specific binding with GFP-ROP1 in Arabidopsis pollen tubes. Table S2. The information of 32 screened mutants from the CS27943 library. Figure S1: Analysis of expression levels of top 400 ROP1-associated genes in different tissues of Arabidopsis. Figure S2: The T-DNA insertion site and DNA genotyping data with identified primers for 4 T-DNA mutants background. Figure S3: The identified 35 ROP1 interactors from the STRING database.
