# Supplementary figures and images for: Rho GTPase ROP1 Interactome Analysis Reveals Novel ROP1-Associated Pathways for Pollen Tube Polar Growth in Arabidopsis
Source: Int J Mol Sci. 2020 Sep 24;21(19):7033. doi: 10.3390/ijms21197033 (PMC7582345; doi:10.3390/ijms21197033)

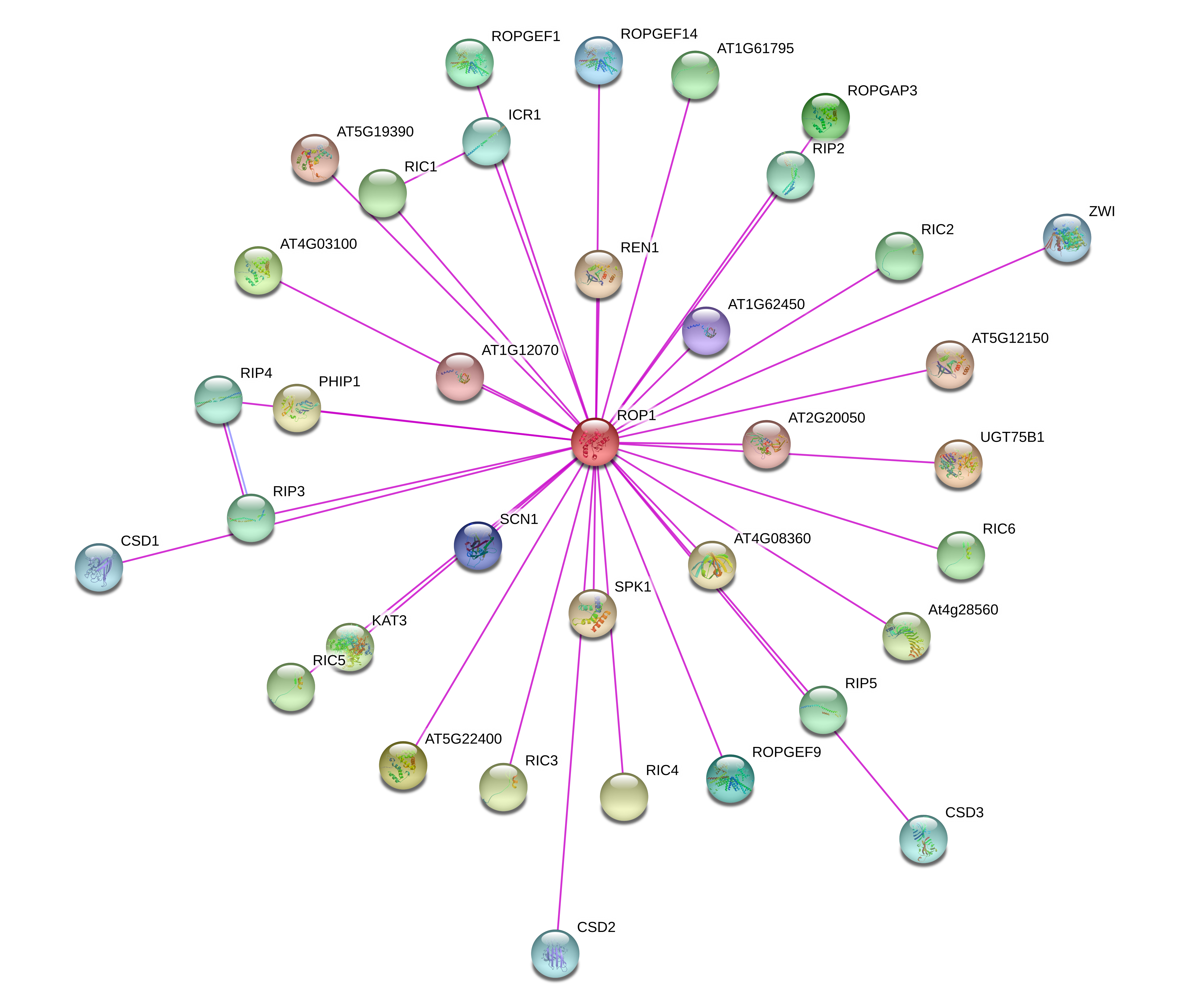

Supplement: Supplementary file 1 [file ijms-21-07033-s001.zip › Fig S1.tif]
